# Modified Epoxy Resin Synthesis from Phosphorus—Containing Polyol and Physical Changes Studies in the Synthesized Products

**DOI:** 10.3390/polym11122116

**Published:** 2019-12-16

**Authors:** Jeong Beom Jang, Tae Hee Kim, Taeyoon Kim, Hye Jin Kim, Bongkuk Seo, Choong-Sun Lim, Wonjoo Lee

**Affiliations:** The Center for Chemical Industry Development, Korea Research Institute of Chemical Technology, 45, Jongga-ro, Yugok-dong, Jung-gu, Ulsan 44412, Korea; beom3374@krict.re.kr (J.B.J.); kimth@krict.re.kr (T.H.K.); xodbs17@krict.re.kr (T.K.); hyejin@krict.re.kr (H.J.K.); bksea@krict.re.kr (B.S.)

**Keywords:** epoxy resin, phosphorous-containing epoxy, thermal stability, heat release rate

## Abstract

Epoxy resins are commonly used to manufacture the molding compounds, reinforced plastics, coatings, or adhesives required in various industries. However, the demand for new epoxy resins has increased to satisfy diverse industrial requirements such as enhanced mechanical properties, thermal stability, or electrical properties. Therefore, in this study, we synthesized new epoxy resin (PPME) by modifying phosphorous-containing polyol. The prepared resin was analyzed and added to epoxy compositions in various quantities. The compositions were cured at high temperatures to obtain plastics to further test the mechanical and thermal properties of the epoxy resin. The measured tensile and flexural strength of epoxy compositions were similar to the composition without synthesized epoxy resin. However, the heat release rates of the compositions exhibited tendencies of a decrease proportional to the amount of PPME.

## 1. Introduction

Epoxy resins are commonly used to manufacture molding composites, reinforced plastics, coatings, and adhesives required for various industrial applications due to their high moisture resistance, mechanical properties, chemical resistance, or thermal properties [1,2,3,4,5,6]. The epoxy composition comprised an epoxy resin and amine or anhydride curer combined to form epoxy three-dimensional polymers useful for these applications. While several studies have focused on improving the mechanical properties of epoxy resins by modifying them or adding tougheners in epoxy compositions [7,8,9,10], the increase of thermal stability related to flame retardant (FR) is an increasing demand in automobiles, aircrafts, or flip-chip encapsulation [11,12,13].

Traditionally, inorganic additives are often used to improve thermal stability such as aluminum hydroxide, magnesium hydroxide, or ammonium polyphosphate (APP). However, for a few applications such as those involving the resin transfer molding system, the modification of epoxy resins is preferred for the uniform distribution of the resin in the composites [12]. An example of a high thermal stability of resin is tetrabromobisphenol A epoxy resin (TBBPA). However, its usage is limited by the toxicity of the resin, which violates the environmental regulations. For the halogen free alternatives, phosphorous-containing organic compounds such as 9, 10-Dihydro-9-oxa-10-phosphaphenanthrene-10-oxide (DOPO), DOPO-hydroquinone (DOPO-HQ), or DOPO- naphthaquinone (NQ) are often used for the epoxy compositions [14]. However, the increase in phosphor content of the compounds essential for high FR is a difficult issue. For example, P content of DOPO is approximately 9.5% while P content of APP is approximately 28%. In other words, more DOPO should be added in the epoxy compositions to ensure that the P content is similar to that of the APP added compositions. Furthermore, it is important to maintain the mechanical properties of epoxy compositions prepared with thermally stable resins.

Here, we synthesized a thermally stable epoxy modified resin and studied the variations in its physical properties for different epoxy compositions. To this end, tensile and flexural strength, and impact strength were measured to test the mechanical properties of the epoxy resins. Differential scanning calorimetry (DSC) and micro combustion calorimetry (MCC) were used to analyze the thermal properties of the epoxy resins.

## 2. Experiments

### 2.1. Materials

Diglycidyl ether of bisphenol F (DGEBF, Epikote 862, epoxy equivalent weight (EEW, g/eq unit), 177 g/eq), and diglycidyl ether of bisphenol A (DGEBA, Epikote 828, EEW 187 g/eq) were obtained from Momentive Co. (Seoul, Korea). D-230 (average molecular weight: 230 g/mol) was obtained from Kukdo Chemicals (Seoul, Korea). Ethylene glycol (g/mol), tetrabutylammonium iodide (TBAI), trimethylamine (g/mol), phenylphosphonic dichloride (PPD) (g/mol), methylene chloride, and diethyl ether were purchased from Sigma-Aldrich (St. Louis, MO, USA).

### 2.2. Synthesis of Phosphor-Containing Polyol (P-Polyol)

**Figure 1:**
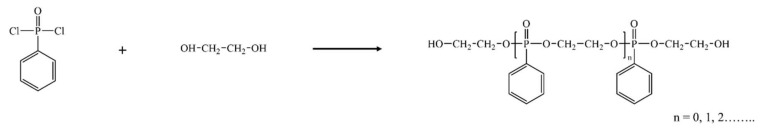
Reaction scheme of polyol synthesis.

**Figure 2:**
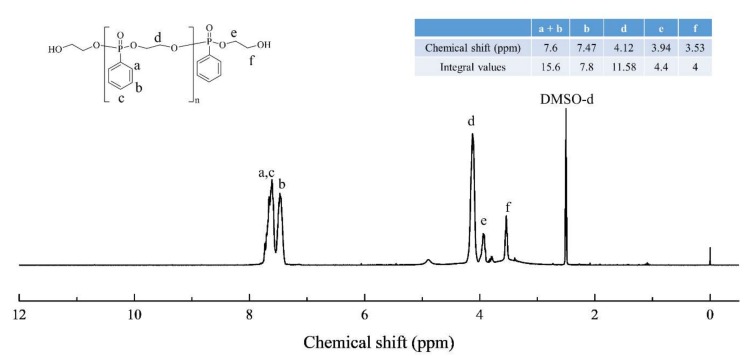
^1^H-NMR data of produced polyol.

**Figure 3:**
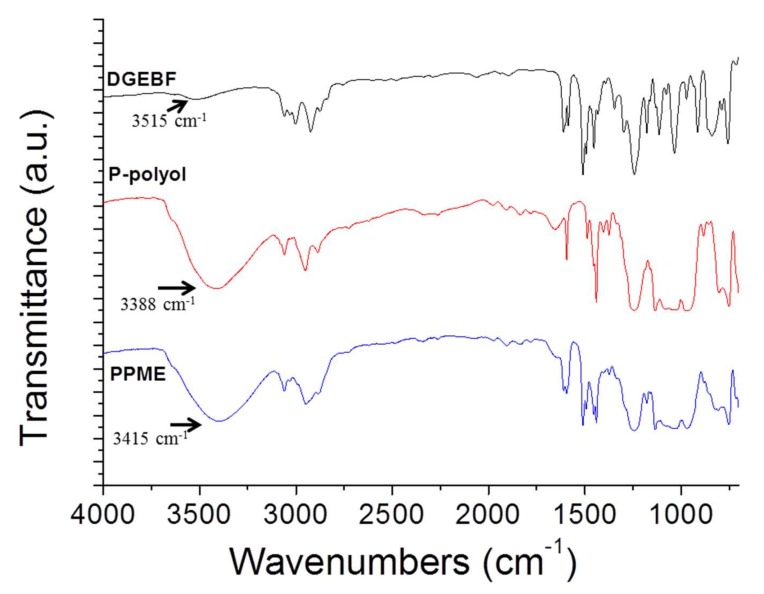
FT-IR spectrum of DGEBF, P-polyol, and PPME.

Ethylene glycol (20 g), and trimethylamine (60 g) were added in a 250 mL three neck round bottom flask filled with 30 mL of methylene chloride. PPD (55 g) in 100 mL of methylene chloride was slowly added using a dropping funnel into the flask and stirred for 24 h at room temperature (Figure 1), and then the resulting solution was filtered to separate solid residues. Deionized water was poured into the resulting solution in a separation funnel. This procedure was repeated several times. The product in methylene chloride was dried at 40 °C for 24 h (Figure 1). The product was cooled to room temperature followed by analysis via ^1^H-NMR (Figure 2) and FT-IR (Figure 3) to confirm that the reaction was completed. The molecular weight of the P-polyol was calculated by converting the –OH value observed by the titration method. The NMR (Bruker Avance 300 spectrometer, Bruker) analysis was conducted in DMSO-d_6_ solvent, ^1^H NMR (300 MHz, DMSO-d_6_): δ = 7.8~7.4 (aromatic H), 4.2~4 (4H, PO-CH2CH2-OP), 3.9~3.8 (2H, O-CH2CH2-OH), 3.6~3.4 (2H, O-CH2CH2-OH).

### 2.3. Synthesis of P-Polyol Modified Epoxy (PPME)

**Figure 4:**
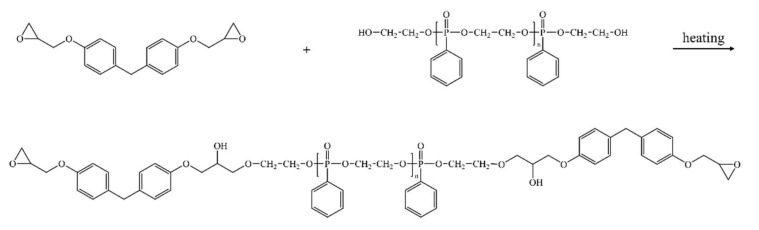
Reaction scheme of PPME.

P-Polyol Modified Epoxy (PPME) was synthesized by reacting P-polyol (76 g, 0.075 mol) with bisphenol F epoxy resin (*M*_W_. 370 g, 56 g, 0.15 mol) with the presence of tetrabutylammonium iodide in 250 mL of the reactor (Figure 4). The solution was stirred at 120 °C for 1 h to evaporate out the filtrate, leaving us with the product. The obtained liquid was analyzed with the ASTM D 1652 method to calculate the epoxy equilibrium weight (EEW).

### 2.4. Preparation of the Epoxy Compositions Including PPME and Its Curing

DGEBA and D-230 were stoichiometrically mixed considering their equivalent molar ratio (Equation (1)). The amount of synthesized PPME in the compositions was varied in the range from 5 to 15 parts by weight per 100 parts of epoxy resin (phr). The components of the epoxy compositions are summarized in Table 1.

{100 g (of epoxy resin)}/{187 (g/eq)} × 60 (amine hydrogen equivalent weight (AHEW)(1)

The compositions were prepared by mixing DGEBA, PPME, and D-230 and stirred with mechanical stirrer for 30 min at 40 °C under vacuum. To prepare the cured plastic test specimen, the paste was poured into a metal mold. The polymerization between epoxy resin and D-230 was preceded by heating the mold for 1 h at 80 °C in a heating oven.

### 2.5. Measurements and Analyses

The synthesized P-polyol was analyzed using proton nuclear magnetic resonance (^1^H-NMR, Bruker Avance 300 MHz spectrometer, Bruker, MA, USA) spectroscopy in the DMSO-d6 solvent. The molecular weight of P-polyol was obtained by titrating the –OH value with a potentiometric titrator (Metrohm 888 Titrando, Metrohm AG, Herisau, Swiss) with the ASTM E 1899-08 method. For the experiment, the hydroxyl group in the polyol was reacted with an excess amount of toluene-4-sulfonyl-isocyanate to form carbamate. The carbamate was titrated with tetrabutylammonium hydroxide to obtain the hydroxyl value. Furthermore, P-polyol, PPME, and F epoxy were analyzed using Fourier transform-infrared spectrometry (FT-IR, Nicolet 6700/Nicolet Continuum; Thermo Fisher Scientific Inc., Walthum, MA, USA). The thermal properties of the compositions were observed using differential scanning calorimetry (DSC, Q2000, TA Instruments, New Castle, DE, USA) by running DSC from 25 °C to 250 °C at a heating rate of 10 °C/min. The heat resistance was studied with pyrolysis combustion flow calorimetry (PCFC, Fire Testing Technology Limited, West Sussex, UK) with the ASTM D7309 method. A sample loaded in the instrument is heated at a rate of 1 °C/min from 100 to 900 °C to obtain heat release rate (HRR) and total heat release (THR). The mechanical properties of the cured epoxy compositions in the metal mold were tested by processing the plastics to achieve a 60 × 25 × 3 mm test specimen for flexural strength tests by the ASTM D790M standard and of 150 × 13 × 3 mm for the tensile strength test by the ASTM D638 method. Both experiments were conducted using a universal testing machine (UTM 5982, Instron, MA, USA). The impact strength was measured with an Izod impact tester (JJHBT-6501, JJ-test, Chengde, China) by the ASTM D256 method, while viscoelastic properties of the cured compositions were evaluated by dynamic mechanical analysis (DMA, Q800, TA Instruments, Inc., New Castle, DE, USA) processed at a size of 60 × 12 × 3 mm. DMA experiments were conducted by mounting the test samples on a dual cantilever probe and tested at a heating rate of 5 °C/min from 25 to 200 °C at a frequency of 1 Hz. The fractured surface was observed with the obtained samples during the impact test by FE-SEM (MIRA 3, TESCAN, Brno, Czech Republic). Epoxy equivalent weight (EEW) was measured by the ASTM D 1652 method.

## 3. Results and Discussion

### 3.1. Structural Characterization of P-Polyol and PPME

The synthesized P-polyol was analyzed with ^1^H-NMR (Figure 2). The peaks at 7.8–7.4 ppm corresponded to phenoxyl protons while the peaks at 4.2–4.0 ppm belonged to ethoxyl protons. The obtained –OH value of the polyol from potentiometric titration was 110.7 mg KOH/g, and the converted molecular weight was 1013.6 g/mol. The FT-IR spectra of DGEBF, P-polyol, and PPME are displayed in Figure 3. The small intensity of the peak at 3519 cm^−1^ was assigned to the epoxy hydroxyl peak in DGEBF. The peak intensity considerably increased due to the reaction with P-polyol, resulting in PPME. The hydroxyl peak of P-polyol at 3388 cm^−1^ shifted to 3415 cm^−1^ upon the reaction (Figure 3). The epoxy hydroxyl peak of PPME at 3415 cm^−1^ appeared at a slightly lower wavelength number with that of DGEBF; however, the peak intensity considerably increased. Furthermore, the EEW of PPME was measured using the ASTM D1652 method and confirmed to be 964 g/eq. This suggests that the reaction was slightly over reacted because the ideal molecular weight is 876 g/eq.

### 3.2. Cure of the Epoxy Compositions and Thermal Properties of Epoxy Polymer

**Figure 5:**
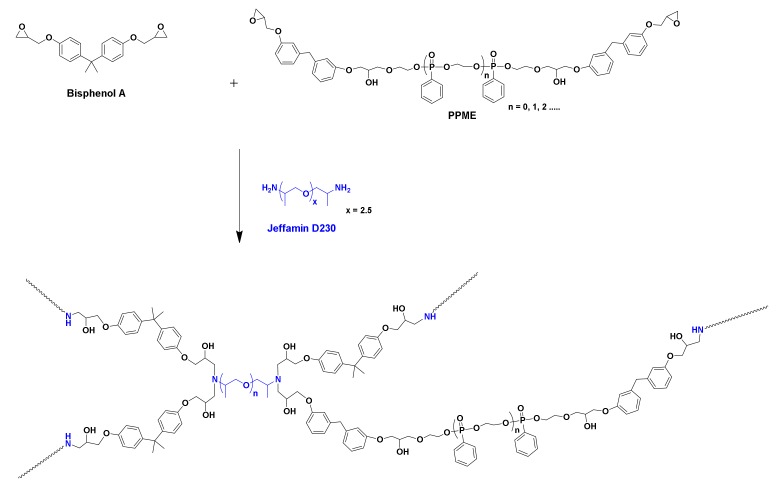
Reaction scheme among bisphenol A epoxy resin, PPME, and D-230.

**Figure 6:**
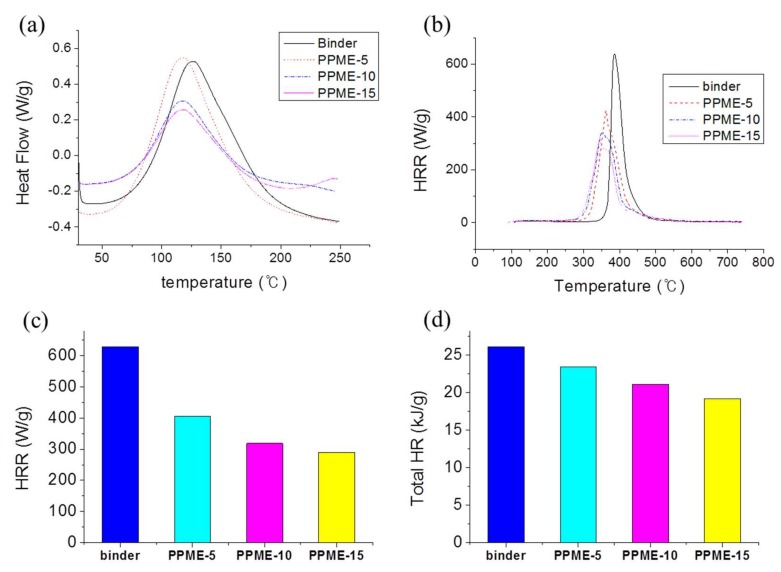
(**a**) DSC curves of epoxy compositions), (**b**) HRR vs. temperature curves, (**c**) HRR data, (**d**) THR of the epoxy compositions with PPME.

The reaction scheme of bisphenol A epoxy resin, PPME, and D-230 is displayed in Figure 5. A proton of amine reacts to one epoxy group, leading to the formation of networked epoxy polymers. The reactions of the compositions were monitored to set curing temperatures. As displayed in Figure 6a, all compositions exhibited a similar onset temperature (T_onset_) of 77 °C and peak temperature (T_peak_) of 116 °C. As there is no significant variation due to the added P-polyol, all samples were cured at 80 °C, which is slightly higher than T_onset_ to provide sufficient energy for the polymerization.

Thermal properties of the cured epoxy compositions with PPME were studied with micro combustion calorimetry (MCC) (Figure 6b) by measuring the heat release rate (HRR, Figure 6c), peak temperature (T_peak_), and total heat release (THR, Figure 6d). The obtained data are organized in Table 2. The HRR value of the epoxy compositions exhibited a tendency to decrease from 628.9 for the binder to 289.9 W/g for PPME-15 proportional to the increase of PPME. Additionally, the peak temperature increased with PPME. Furthermore, the trend of THR value exhibited the same pattern of decrease from 26.1 kJ/g (binder) to 19.2 kJ/g (PPME-15), indicating that the heat resistance is affected to the amount of PPME with phosphorous.

### 3.3. Mechanical Properties of the Cured Epoxy Compositions

**Figure 7:**
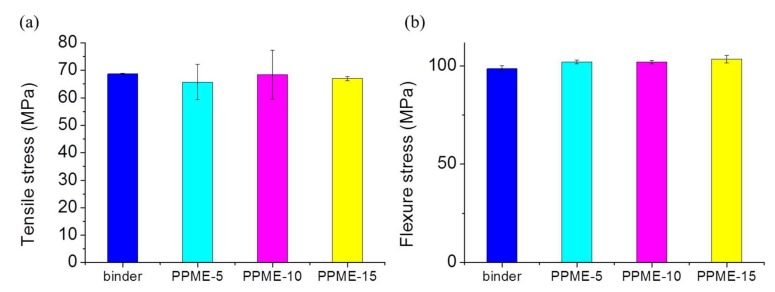
Tensile (**a**) and flexural (**b**) strength of the cured compositions.

Mechanical properties such as tensile and flexural strengths of the cured epoxy compositions were analyzed using the UTM (Figure 7). Though it is important to introduce thermally stable additives in epoxy composition, it is also carefully monitored whether the additives deteriorate the physical properties of epoxy compositions. Therefore, tensile and flexural strength experiments of the polymerized epoxy plastics were conducted (Figure 7). Figure 7a shows that the strength was similar for all cases, irrespective of the amount of PPME. Moreover, the flexural strength results also demonstrate that the strength of all samples was similar, indicating that PPME does not lower the physical properties of the produced epoxy polymers.

**Figure 8:**
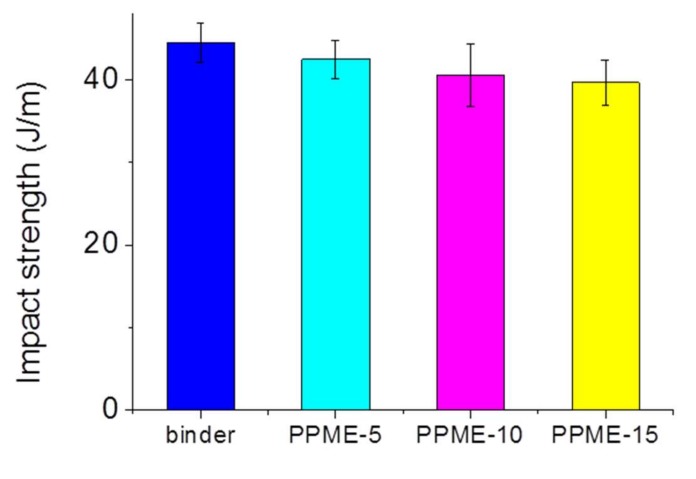
Impact strength of the compositions.

For the Izod impact strength measurement, though the test values of the test specimen exhibited a decreasing tendency, the degree of impact strength was not as noticeable (Figure 8).

**Figure 9:**
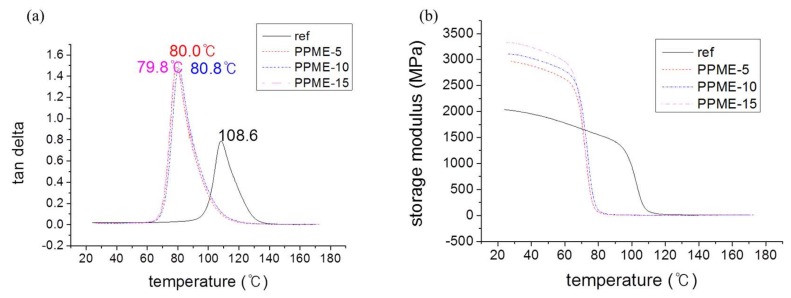
DMA curves, (**a**) tan curves, (**b**) storage modulus curves of epoxy polymers.

The viscoelastic properties of the cured polymers were analyzed with DMA as shown in Figure 9. The curves of tan δ of cured epoxy plastics demonstrate that there was a reduction in the glass transition temperature (T_g_) for the samples with PPME by 20 °C (Figure 9a). The reduction of Tg was partially from the flexible alkyl backbone of PPME. Furthermore, the degree of drops of T_g_s was similar for all samples, irrespective of the amount of PPME. Furthermore, the storage modulus of the samples with PPME increased from 2035 to over 3330 MPa (Figure 9b), indicating the mechanical properties of the cured plastics were enhanced by the addition of PPME.

### 3.4. FE-SEM Images of the Epoxy Polymers

**Figure 10:**
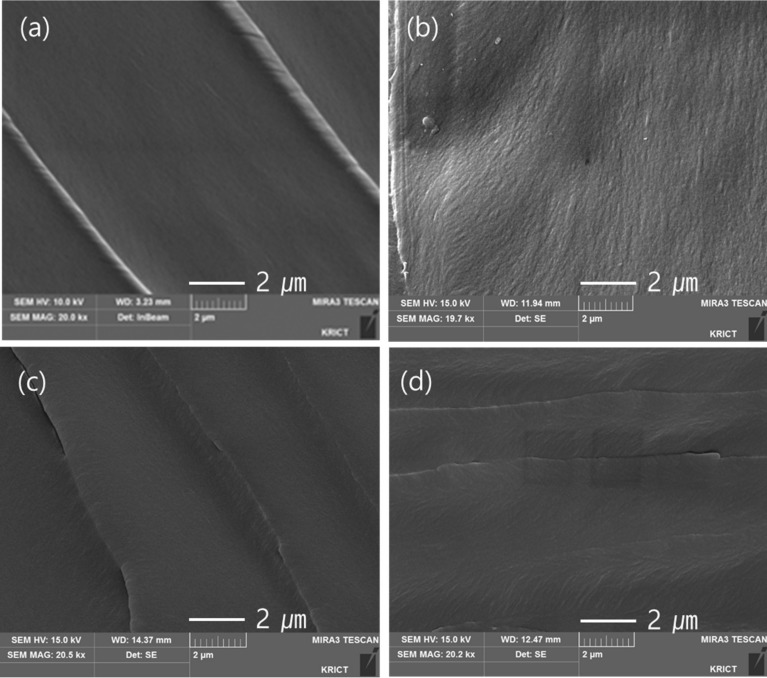
FE-SEM images of the fractured surfaces: (**a**) binder, (**b**) PPME-5, (**c**) PPME-10, (**d**) PPME-15.

The fractured images of the binder and epoxy compositions with PPME obtained from the Izod impact test were observed with FE-SEM (Figure 10). The surface of binder was smooth, and lines formed by impact were clean. However, the lines on the surface of PPME-10 were complex and formed twigs perpendicular to the direction of the line, suggesting that the cured epoxy polymer modified the epoxy polymers.

## 4. Conclusions

In this study, a phosphor-containing polyol was reacted with epoxy resin to provide a modified epoxy resin, PPME. Various amounts of PPME were blended with the mixture of DGEBA and D-230 to afford epoxy compositions. The prepared epoxy compositions were cured at high temperatures to form the epoxy polymer. The variation in the physical properties of the cured plastics with PPME were determined via the tensile, flexural, and impact strength measurements. The cure behavior was monitored using DSC to set cure temperature to 80 °C. The results of tensile and flexural strength demonstrate that the epoxy compositions with PPME remained the same, irrespective of the contents of PPME. Tensile strength was maintained at approximately 68 MPa while flexural strength was at approximately 101 MPa for all test samples in the range of standard deviation. However, the impact strength of the compositions proportionally decreased from 44.5 J/m with the binder to 39.7 J/m with PPME-15. Furthermore, the storage modulus of the cured samples increased from 2035 MPa up to 3330 MPa with the increase in PPME. However, the T_g_ of epoxy compositions tends to be lowered from 108.6 °C to 79.8 °C by the increased amount of PPME. The effect of thermal resistance by adding PPME was monitored with MCC. HRR decreased from 629 W/g with the binder to 290 W/g with PPME-15, while the total heat release decreased from 26.1 kJ/g with the binder to 19.2 kJ/g with PPME-15.

## Figures and Tables

**Table 1:** Formulation of the epoxy compositions.

Components (g)	Reference	PPME-5	PPME-10	PPME-15
DGEBA	100 (26.7 mol)	100	100	100
D-230	32.1 (14.0 mol)	32.1	32.1	32.1
PPME	0	5 (0.49 mol)	10 (0.99 mol)	15 (1.5 mol)

**Table 2:** Thermal data obtained by MCC experiments.

	Binder	PPME-5	PPME-10	PPME-15
HRR (W/g)	628.9	406.7	318.7	289.9
Total HR (kJ/g)	26.1	23.4	21.1	19.2

## References

[1] Gamardella F., Sabatini V., Ramis X., Serra A. (2019). Tailor-made thermosets obtained by sequential dual-curing combining isocyanate-thiol and epoxy-thiol click reactions. Polymer.

[2] Chu W.C., Lin W.S., Kuo S.W. (2016). Flexible epoxy resin formed upon blending with a triblock copolymer through reaction-induced microphase separation. Materials.

[3] Greiner L., Kukla P., Eibl S., Döring M. (2019). Phosphorus containing polyacrylamides as flame retardants for epoxy-based composites in aviation. Polymers.

[4] Zhang C., Dai X., Wang Y., Sun G., Li P., Qu L., Sui Y., Dou Y. (2019). Preparation and corrosion resistance of ETEO modified graphene oxide/epoxy resin coating. Coatings.

[5] Cheng Y., Zhang Q., Fang C., Chen J., Su J., Xu K., Ai L., Liu D. (2018). Preparation, structure, and properties of surface modified graphene/epoxy resin composites for potential application in conductive ink. Coatings.

[6] Bian X., Tuo R., Yang W., Zhang Y., Xie Q., Zha J., Lin J., He S. (2019). Mechanical, thermal, and electrical properties of bn–epoxy composites modified with carboxyl-terminated butadiene nitrile liquid rubber. Polymers.

[7] Giannakopoulos G., Masania K., Taylor A.C. (2011). Toughening of epoxy using core–shell particles. J. Mater. Sci..

[8] Liu S., Fan X., He C. (2016). Improving the fracture toughness of epoxy with nanosilica-rubber core-shell nanoparticles. Compos. Sci. Technol..

[9] Kim T., Kim S., Lee D., Lim C.S., Seo B. (2018). Preparation of a branched amine and the physical and thermal studies of the epoxy compositions including the amine compound. J. Appl. Polym. Sci..

[10] Tsang W.L., Taylor A.C. (2019). Fracture and toughening mechanisms of silicaand core–shell rubber-toughened epoxy at ambient and low temperature. J. Mater. Sci..

[11] Mariappan T., Wilkie C.A. (2014). Flame retardant epoxy resin for electrical and electronic applications. Fire Mater..

[12] Wang N., Teng H., Yang F., You J., Zhang J., Wang D. (2019). Synthesis of K-carrageenan flame-retardant microspheres and its application for waterborne epoxy resin with functionalized graphene. Polymers.

[13] Wang N., Teng H., Zhang X., Zhang J., Li L., Zhang J., Fang Q. (2019). Synthesis of a carrageenan–iron complex and its effect on flame retardancy and smoke suppression for waterborne epoxy. Polymers.

[14] Khalifah A., Salmeia S.G. (2015). An overview of some recent advances in DOPO-derivatives: Chemistry and flame retardant applications. Polym. Degrad. Stab..

